# Supplementary posterior fusion in patients operated on employing TLIF may decrease the instrumentation failure rate

**DOI:** 10.3389/fsurg.2023.1259946

**Published:** 2023-12-22

**Authors:** Andrey Bokov, Svetlana Kalinina, Mingiyan Khaltyrov, Svetlana Pavlova, Anatoliy Bulkin

**Affiliations:** ^1^ Institute of Traumatology and Orthopedics, Privolzhsky Research Medical University, Nizhny Novgorod, Russia; ^2^ Department of Neurosurgery, Institute of Traumatology and Orthopedics, Privolzhsky Research Medical University, Nizhny Novgorod, Russia

**Keywords:** transforaminal lumbar interbody fusion, circumferential fusion, posterior fusion, degenerative diseases, lumbar spine, screw loosening

## Abstract

**Background:**

It is supposed that additional posterior fusion may provide additional stability of the pedicle screw; however, the clinical impact of additional posterior fusion in patients treated with TLIF remains uncertain. The objective of this study is to assess the clinical efficacy of circumferential fusion in patients treated with TLIF.

**Materials and methods:**

This is a single-center retrospective evaluation of consecutive 179 patients with degenerative lumbar stenosis and instability of spinal segments. Patients with axial pain and neurogenic claudication or radiculopathy associated with spinal stenosis were enrolled during the period from 2012 to 2018. Transforaminal lumbar interbody fusion (TLIF) with a single cage was used to treat patients. In 118 cases a supplementary posterior fusion was made. The duration of follow-up accounted for 24 months, logistic regression analysis was used to assess factors that influence the complication rate.

**Results:**

The rate of pedicle screw loosening was growing with radiodensity getting decreased and was more frequent in patients with two level fusion. An increase in pedicle screw loosening rate correlated with anterior nonunion Tan 2 and 3 grade while both posterior complete and incomplete fusion resulted in a decline in the complication rate. Lumbosacral fusion, bilateral facet joints` resection and laminectomy turned out to be insignificant factors. The overall goodness of fit of the estimated general multivariate model was *χ*^2^ = 87.2230; *P* < 0.0001. To confirm clinical relevance of those findings, a univariate logistic regression was performed to assess the association between clinically significant pedicle screw instability and posterior fusion in patients operated on employing TLIF. The results of logistic regression analysis demonstrate that additional posterior fusion may decrease the rate of instrumentation failure that requires revision surgery in patients treated with TLIF [B0 = 1.314321; B1 = −3.218279; *p* = 0.0023; OR = 24.98507; 95% CI (3.209265; 194.5162), the overall goodness of fit of the estimated regression was *χ*^2^ = 22.29538, *p* = <0.0001].

**Conclusion:**

Circumferential fusion in patients operated on employing TLIF is associated with a decline in the rate of pedicle screw loosening detected by CT imaging and clinically significant instrumentation failure.

## Introduction

Being one of the most common causes of disability, spinal stenosis of the lumbar spine is a frequently encountered morbid condition in the elderly adult population (1). In cases where spinal stenosis is associated with instability of the affected segments, decompression and stabilization employing various types of fusion are required to achieve clinically significant results, and with an aging population, the number of cases operated on annually keeps on growing (2, 3).

Pedicle screw fixation with transforaminal interbody fusion is a common technique that is applied to treat patients with spinal stenosis and instability. The discussed approach provides a direct decompression that can be used in all cases of lumbar stenosis, while pedicle screw fixation and interbody fusion provide long-term stability (4, 5). Despite the reported efficacy, interventions employing pedicle screw fixation and fusion have a certain rate of complications, and the most frequently reported are pedicle screw loosening and pseudoarthrosis (6–8). Altered bone quality, which has a considerable prevalence in the elderly adult population, was proven to be the most contributing factor to those complications' development (9–11). Different tools are used to detect patients who are at risk of instrumentation failure development, and measurements of radiodensity in Hounsfield units became popular because it correlates with the mechanical properties of bone and the rate of pedicle screw loosening and pseudoarthrosis consequently (12).

Various strategies were proposed to reduce implant-dependent complication rates after spinal instrumentations. Out of those, the most frequently used are the application of a broad cage, augmentation of vertebral bodies, and various alterations of pedicle screw design suggested to increase the strength of pedicle screw purchase in cancellous bone (13, 14). However, the application of those options is material and cost-consuming and has certain technical limitations and risks. To achieve higher rate of fusion, a circumferential fusion was suggested in patients treated with TLIF. The recommended technique appears to be less technically demanding than fusion from anterior approaches, and it is not associated with additional morbidity, however neither superiority no inferiority towards other techniques was proven (4, 5, 15). It is supposed that additional posterior fusion may provide additional stability; however, the clinical impact of additional posterior fusion in patients treated with TLIF remains uncertain (15).

The objective of this study is to assess the clinical efficacy of circumferential fusion in patients treated with TLIF.

### Materials and methods

This study is a single-center retrospective evaluation of consecutive 179 patients with degenerative lumbar stenosis and instability of spinal segments including 51 male and 128 females. The age of participants at the time of operation was *M* (median) = 52 years [25%–75% (44; 76); range 19–80 years]. Patients with axial pain and neurogenic claudication or radiculopathy associated with spinal stenosis were enrolled. Participants underwent spinal instrumentations employing pedicle screw fixation with transforaminal interbody fusion during the period from 2012 to 2018. The duration of follow-up accounted for 24 months. Radiographic criteria of pedicle screw loosening were used to assess outcomes. This study was reviewed and approved by the local institutional board committee, given that no additional risks were anticipated.

The inclusion criteria were:

• Presence of degenerative lumbar spinal stenosis with unstable spinal segments confirmed by functional radiograms or low-grade symptomatic unstable spondylolisthesis

Indications for spinal instrumentation were:

• Neurological deficit associated with spinal stenosis,
• Neurogenic claudication,
• Axial and radicular pain syndromes with visual analog scale (VAS) over 4 (0–10) and Oswestry Disability Index (ODI) over 40% resistant to repeated conservative treatment during 3 months or neurogenic claudication

The exclusion criteria were:

• High-grade spondylolisthesis (grades 3 and 4),
• Degenerative deformities that required fixation of more than 5 segments or spinopelvic fixation, sagittal and frontal imbalance and spinopelvic parameter mismatches that require more than 5-segment fixation and spinopelvic fixation,
• Tumor-related lesions of the lumbar spine,
• Revision surgery,
• Cases with screw malposition and redirection detected on postoperative CT images,
• Patients with different types of fusion applied on different levels,
• Cases operated on more than two levels.

Before the procedure, all patients underwent dynamic x-ray imaging with flexion and extension and CT examination. The criterion for spinal instability was anterior translation greater than 3 mm detected on dynamic x-ray (16). CT scans were performed using a single CT scanner (Aquilion 32, Toshiba Corporation). Standard protocol was used during all examinations: slice thickness of 0.5 mm, covering a scan area of 50 cm, tube voltage and current were 120 kV and 300 mA respectively, auto mAs range 180–400, helical-pitch 21.0. Integrated software was used to assess the results of CT (Vitrea Version 5.2.497.5523) with a window width/window level ratio of 2,000/500. During CT examinations, measurements of a vertebral body cancellous bone radiodensity in HU were obtained at standard level of L3 in the sagittal, axial, and coronal planes. CT investigation was performed by two independent certified radiologists. Measurements in the axial plane were taken at the level of the middle of the pedicles while those in the sagittal and coronal planes were taken along the geometric center of the vertebral body. Trabecular bone samples were selected using the maximal achievable square without traversing into cortical bone. Out of those three measurements, an average radiodensity was calculated for each case.

Transforaminal lumbar interbody fusion (TLIF) with a single cage was used to treat all the enrolled patients in this study. The applied technique of TLIF was standard open procedure. The TLIF approach was performed from side corresponding neurological presentations, finally unilateral facet joints removal was performed to achieve exposure sufficient for disc removal and cage placement. If bilateral decompression was required, the crossover decompression was done employing partial resection of the facet joints on the opposite side. In 118 cases out of 179 (65.9%) a supplementary posterior fusion was made using standard technique. Firstly, a decortication of articular processes was performed, then facet joints cartilages with the adjacent bone were removed and the gap formed was filled up with autologous bone with the additional bone placed on decorticated articular processes. Autograft of locally harvested bone during decompression was used to perform TLIF and posterior fusion. Bilateral open pedicle screw fixation employing polyaxial screws was used in all cases, the applied technique was standard; strait trajectory for screw placement was used. Pedicle screws were introduced at least to the anterior third of a vertebral body; bicortical screw placement was not used in the enrolled patients. The qualification of surgeon was at least 7 years of experience.

The duration of the follow-up accounted 24 months. All patients underwent clinical examination at the time of 3, 6, 12, 24 months. CT examinations was performed at the time of 6, 12, months after surgery and regardless time period if clinical signs of implant failure signs were detected. During CT examination anterior and posterior fusion was assessed.

According to the results of CT anterior fusion was classified as:

• Bipolar fusion—no radiolucent zone detected between bone graft, upper and lower endplate with an evident bipolar bone bridging,
• Unipolar pseudoarthrosis—radiolucent zone detected between bone graft and one of the endplates,
• Complete pseudoarthrosis—a radiolucent zone detected between.

Posterior fusion was classified as:

• Complete fusion—evidence of trabeculation, complete ankylosing of facet joint,
• Partial fusion—bone bridging between articular processes present, however only partial ankylosing was achieved,
• Total non-union—no bone bridging detected.

**Figure 1:**
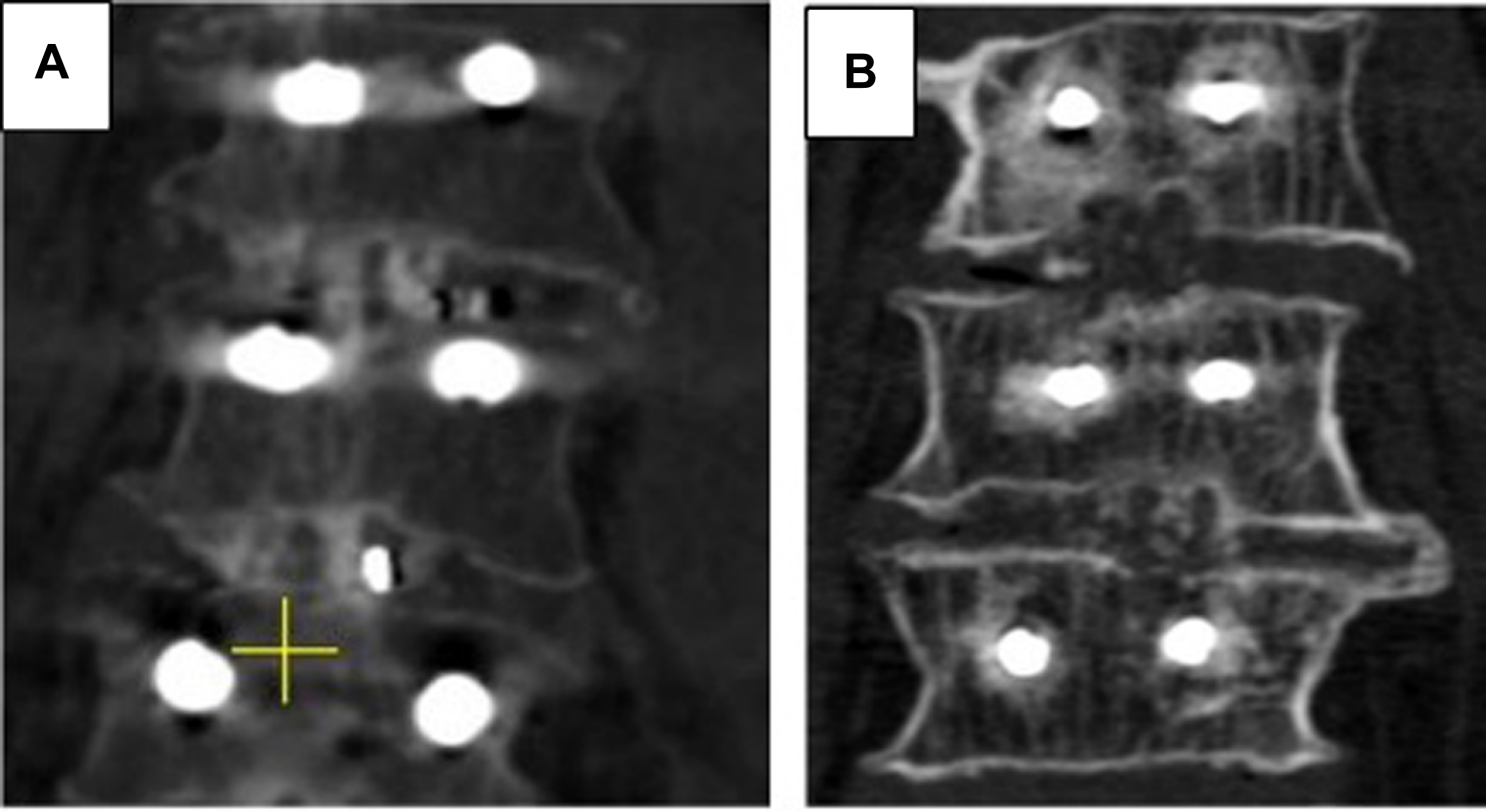
Grades of anterior fusion. (**A**) CT scan in coronal plane, 1 year after surgery, bipolar bridging was confirmed on both levels. (**B**) CT scan in coronal plane, 1 year after surgery, total non-union was detected on the upper operated and unipolar pseudoarthrosis – on the lower level.

**Figure 2:**
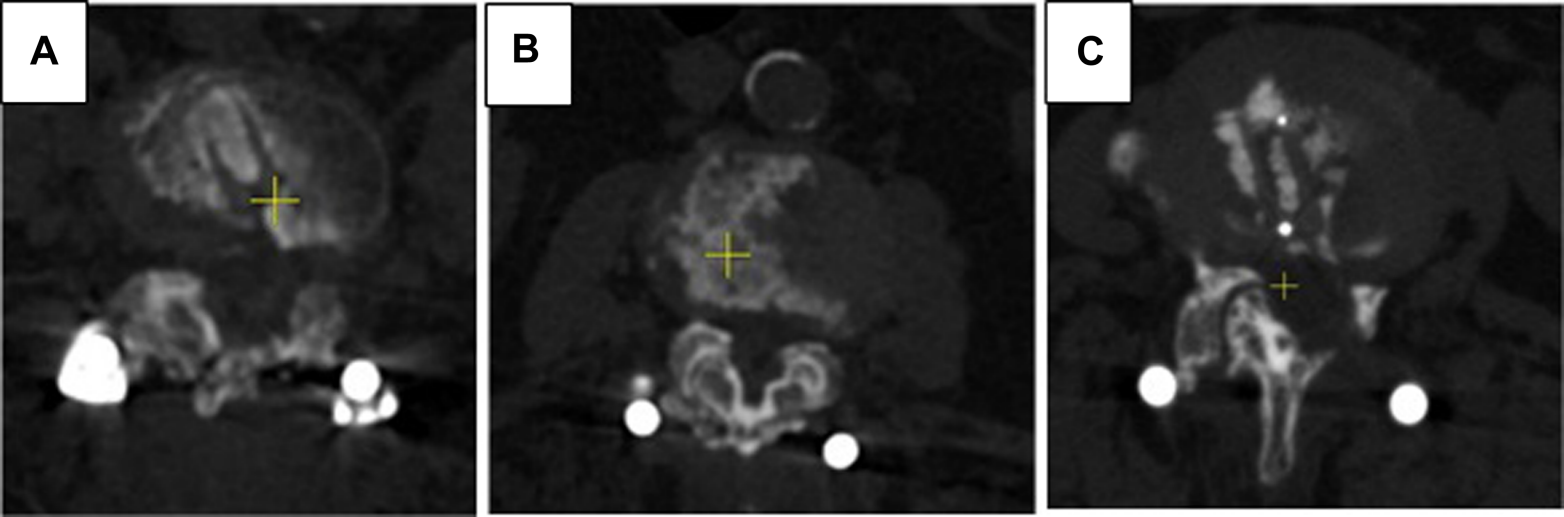
Grades of posterior fusion. (**A**) CT scans in axial plane, 1 year after surgery - total ankylosing of facet joints was confirmed. (**B**) CT scans in axial plane, 1 year after surgery - partial ankylosing of facet joints was confirmed. (**C**) CT scans in axial plane, 1 year after surgery - complete posterior nonunion was detected.

Grades of anterior and posterior fusion according to the results of CT examination are given on Figures 1, 2 respectively.

The criterion for screw loosening was a 1-mm or greater radiolucent zone around the screw, double-halo sign, or both (17). Finally, patient outcomes were classified as either presence of pedicle screw loosening signs, regardless the number of screws loosened, or absence of this complication. Cases with pedicle screw loosening were subdivided into clinically significant and asymptomatic ones.

### Statistical analysis

Fisher`s exact test was used to estimate statistical significance of the observed differences in rate of pedicle screw loosening and revision surgeries applied. The association between screw loosening rate and potential risk factors was assessed using logistic regression analysis (general multivariate logistic regression model of the highest explanatory value). The following software was used for statistical analysis: Statistica 12, SPSS 22.0.

## Results

The characteristics of the enrolled group of patients are given in Table 1. A statistically significant difference in age was detected between groups treated with TLIF and those who underwent circumferential fusion (*M* = 49 years (25%–75% [44; 76]; range 19–76 years vs. *M* = 54, [25%–75% (46; 62); range 20–80 years respectively, *p* = 0,0339, Mann–Whitney test was used]. By the end of the follow-up period, CT signs of pedicle screw loosening were detected in 54 patients (30.2%) patients, out of those 19 (10.6%) deteriorated with axial pain VAS of more than 4 and ODI scores over 40; the latter 19 patients underwent revision surgery. Patients with clinically significant instability presented with either multiple pedicle screws instability or bilateral one level screw loosening along with either unipolar or bipolar pseudoarthrosis after interbody fusion and lack of posterior fusion. Relatively high prevalence of CT loosening signs can be explained by considerable proportion of patients with radiodensity below 110 HU that correspond 90% specificity of osteoporosis detection.

**Table 1:** Characteristics of the enrolled group.

Characteristics	Value
Age, years	*M* = 52; 25%–75% [44; 76]; range: 19–80
Male to female ratio	51/128
Radiodensity, HU	132.6464 ± 3.2437 SD = 43.3980 Range 282.0667–43.1667
2 level fusion applied	54 (30.2%)
Patients with radiodensity of cancellous bone below 110 HU	59 (33.0%)

To assess a contribution of surgery and patient- and surgery-related factors to screw loosening rate detected on CT a general logistic regression model of was applied. Those used for analysis were bone density measured in HU, number of fused levels (1 level vs. 2 level instrumentations), the degree of posterior and anterior fusion, the extensiveness of posterior tension band structures resection including laminectomy and bilateral facetectomy. Finally, the logistic regression model with the highest explanatory level was chosen. The parameters of estimated general multivariate logistic regression model with highest explanatory value are present in Table 2.

**Table 2:** Parameters of the estimated logistic regression function.

Components of regression model	Regression coefficient and its statistical significance	OR per unit change with 95% CI
Intercept	1.73931 *p* = 0.1017	
Radiodensity in HU	−0.00222405 *p* = 0.0012	0.978005 [0.9650613; 0.9911223]
Number of levels fused	1.916489 *p* = 0.0002	6.79705 [2.492831; 18.5331]
CT signs of either partial or complete posterior fusion	−1.871099 *p* = 0.0007	0.1539544 [0.05284661; 0.448505]
CT signs of either unipolar posterior nonunion (Tan 3 or Tan 4)	2.055019 *p* = 0.0007	7.806988 [2.364458; 25.77719]
Lumbosacral fusion	0.071055 *p* = 0.8925	1.07364 [0.3807305; 3.02761]
Bilateral facetectomy	0.6827201 *p* = 0.2964	1.979254 [0.5466886; 7.165774]
Laminectomy	−0.6495271 *p* = 0.3386	0.5222927 [0.1373006; 1.986806]

The rate of pedicle screw loosening rate was growing with radiodensity getting decreased and was more frequent in patients with two level fusion. An increase in pedicle screw loosening rate was correlated with anterior nonunion Tan 2 and 3 rate while both posterior complete and incomplete fusion resulted in a decline in pedicle screws loosening rate. Lumbosacral fusion, bilateral facet joints resection and laminectomy turned out insignificant factors. The overall goodness of fit of estimated general multivariate model was *χ*^2^ = 87.2230; *P* < 0.0001. To determine whether those findings have clinical relevance an univariate logistic regression was performed to detect association of clinically significant pedicle screw instability and posterior fusion in patients operated on employing TLIF. The parameters of logistic regression were: B0 = 1.314321; B1 = −3.218279; *p* = 0.0023; OR = 24.98507; 95% CI [3.209265; 194.5162], the overall goodness of fit of estimated regression was *χ*^2^ = 22.29538, *p* = <0.0001. The results of logistic regression analysis demonstrates that additional posterior fusion may decrease the rate of pedicle screw fixation failure in patients treated with TLIF.

### Discussion

Because of the aging population, an evident trend is observed, resulting in a gradual increase in the prevalence of osteoporosis and degenerative diseases of the lumbar spine (1, 18–20). As a consequence, the annual number of operations employing pedicle screw fixation and various types of fusion keeps growing. Despite evident progress in the treatment of the degenerative diseases of the lumbar spine, the reported rate of complications after pedicle screw fixation and spinal fusion remains considerable, and the most frequently reported are pedicle screw loosening and symptomatic pseudoarthrosis (21–24).

It has been clearly defined that both of the mentioned complications are associated with the deterioration of bone quality (25). Out of different options, CT radiodensity measurement became a popular tool for bone quality assessment because it has been proven that it correlates with the mechanical strength of bone (12, 26, 27). As a result, radiodensity correlates with the rate of the previously mentioned complications. On the other hand, no valid models with acceptable accuracy based on a high level of evidence studies were provided. The presumable reason for the lack of a valid model for complications prediction is that a considerable number of factors may impact the results of fusion. As it has been demonstrated, the extension of fusion, type of fusion, surgical technique and type of implant may influence the rate of instrumentation failure (13, 21, 29). For this reason, the group of patients was standardized by the number of levels and type of fusion; thus, only cases with one- or two-level fusion applying TLIF were enrolled.

The TLIF technique is one of the most frequently used to treat patients with degenerative diseases of the lumbar spine. Even though fusion from the anterior approach may provide a favourable distribution of forces resulting in a pedicle screws` load decline, the indirect decompression reached is not always effective, especially in cases with severe spinal stenosis (Schizas D) and in patients with lateral stenosis (28–31). A strong point of the TLIF technique is that an effective decompression can be provided in all cases of spinal stenosis, while weak points can be compensated by the application of screws with optimal parameters (13). Bone augmentation and 360° fusion are also recommended to achieve maximal effectiveness of the discussed technique (32–35). On the other hand, no high-evidence publications are available that provide clinical evidence for the effectiveness of circumferential fusion (15).

In the present study, the impact of additional posterior fusion on pedicle screw loosening rate was assessed. Anterior fusion was graded according to the Tan classification, with the difference that Tan 1 and Tan 2 degrees of fusion were merged because both are not associated with the potential instrumentation failure (36). Posterior fusion was classified as total fusion, partial fusion, or non-union (37). The follow-up period of 24 months proved sufficient time for fusion formation (38). The results of our study demonstrate that even partial posterior fusion may result in a considerable decline in the rate of pedicle screw loosening detected by CT examination. Taking in view that not every case of pedicle screw loosening is clinically significant, the association of posterior fusion with the rate of pedicle screw instrumentation failure that required revision surgery was assessed. It has been estimated that additional partial or total posterior fusion has the potential to decrease the rate of clinically significant complications. The explanation for the observed effect of posterior fusion is that posterior structures could have a higher density because of being less affected by osteoporosis and hypertrophic changes. As a consequence, with being dependent on bone properties, the formation of posterior fusion could be more efficient (39–41).

To address the potential bias, additional variables were included in the general logistic regression model. Those were lumbosacral fusion, one-level vs. two-level fusion, bilateral facetectomy, and laminectomy. It has been demonstrated that the length of fusion may increase the cantilever arm of forces applied to the endpoints of the construct, while anatomic features of the sacrum might predispose to pedicle screw loosening. Laminectomy and bilateral facetectomy may prevent posterior fusion formation and might increase the range of micromovements of the instrumented level, facilitating screw loosening. According to the results of the analysis, all those variables were statistically insignificant.

### Limitations

The authors admit that the present study is limited because it is retrospective, and the results may be affected by the collinearity of some data. Additional factors, like screws` parameters and heterogeneity in age, were not taken into account. On the other hand, the results of the analysis provide sufficient evidence for the conclusions reached, taking into account that patients who underwent circumferential fusion were older.

## Conclusion

Circumferential fusion in patients operated on employing TLIF is associated with a decline in the rate of pedicle screw loosening detected by CT imaging and clinically significant instrumentation failure.

## Data Availability

The original contributions presented in the study are included in the article/Supplementary Material, further inquiries can be directed to the corresponding author.
